# Validation of the Updated GloboDiet Version by Protein and Potassium Intake for the German National Nutrition Monitoring

**DOI:** 10.3390/nu15204418

**Published:** 2023-10-18

**Authors:** Friederike Wittig, Carolin Krems, Ann Katrin Engelbert, Andrea Strassburg

**Affiliations:** 1 Department of Nutritional Behaviour, Max Rubner-Institut (MRI)-Federal Research Institute of Nutrition and Food, 76131 Karlsruhe, Germany; friederike.wittig@mri.bund.de (F.W.); carolin.krems@mri.bund.de (C.K.); 2 Department of Physiology and Biochemistry of Nutrition, Max Rubner-Institut (MRI)-Federal Research Institute of Nutrition and Food, 76131 Karlsruhe, Germany; annkatrin.engelbert@mri.bund.de

**Keywords:** validation, GloboDiet, 24 h recall, biomarkers, potassium, protein, sodium, energy

## Abstract

(1) Background: The German version of GloboDiet, a software for a computer-based assessment of 24 h recalls, was intensively updated. Therefore, validation is required prior to its use in the upcoming data collection within the German National Nutrition Monitoring. (2) Methods: For this purpose, the cross-sectional ErNst study with 109 participants (57 women and 52 men) was conducted. The study provided data on 24 h GloboDiet recalls and 24 h urine samples from the same day. Protein and potassium intake, known as eligible validation markers, were compared to the measured excretion in urine. To assess the agreement between intake and excretion, the following statistical methods were used: Wilcoxon rank tests, confidence intervals, Spearman correlations, and Bland–Altman plots. (3) Results: Overall, the updated German GloboDiet version showed valid estimates of protein intake. Regarding potassium, results were ambiguous and differed depending on the statistical method applied. While the Bland–Altman plot showed a good agreement between 24 h recalls and urine samples for potassium, the correlation was weak, suggesting that 24 h recalls may underestimate true intake. (4) Conclusions: Despite the partly ambiguous results, the updated GloboDiet version linked to the current German Nutrient Database provides valid estimates of nutrient intake.

## 1. Introduction

To record food consumption, various methods are available. The choice of the method potentially affects the results [1,2,3]. There is no method available that measures dietary intake without error [3]. Dietary assessment methods such as 24 h recalls go along with random and systematic errors caused by difficulties in estimating portion sizes and daily variations in food consumption [1,3,4]. Furthermore, the selected food composition database [2,4,5,6,7] and coding system [7] may also influence results. It has also been shown that characteristics of the data collection such as the mode of administration (face-to-face or telephone) or day of the week (weekday or weekend) [8,9,10] can influence study results. Biomarkers may also be affected by a number of factors, such as dietary composition, health or diseases, gastrointestinal microbiome, or genetic factors [11]. To standardise the procedure for conducting food consumption surveys, the European Food Safety Authority (EFSA) developed methodological guidelines for the assessment of food consumption data [12,13]. For the adult population, EFSA recommends 24 h recalls for the dietary assessment in national nutrition surveys. GloboDiet (formerly EPIC-Soft) is a software for conducting standardized 24 h recalls according to the EFSA recommendations [14]. It has previously been used in surveys of the German National Nutrition Monitoring [15,16] and is intended to be used again in the upcoming study, prepared to launch in 2024. GloboDiet was developed in the 1990s by the International Agency for Research on Cancer (IARC) in Lyon (France) [17] and was used in different countries worldwide [14,17,18,19,20,21]. The 24 h recalls conducted with GloboDiet are interviewer-administered, ensure a structured interview procedure as well as documentation [12,17,22] and include different options of quality assurance [23].

In the past, different country-specific versions of GloboDiet have already been validated within the European Prospective Investigation into Cancer and Nutrition (EPIC) study [24,25] and within the European Food Consumption Validation (EFCOVAL) study [9,10,21]. Those validations showed that the software was sufficiently valid to estimate protein and potassium intake. Due to the ever-changing food supply [26,27], the previous German version of GloboDiet had to be extensively updated. During this process, 600 foods were added and 525 deleted. The latter include foods that are no longer available on the German food market, such as various types of fish due to fishing bans. Examples of newly added foods include vegan and vegetarian products such as cereal drinks. The final food list of the German GloboDiet version includes about 2000 food items. Aside from the food list, the measures used to quantify consumed amounts were actualized (standard units, household measures, food shapes, and a picture book including photo series with different portion sizes). For example, the software was extended to account for the increased range of dairy products in mini, small, medium, and extra-large sizes, mini fruits and vegetables such as baby bananas, as well as different sizes of coffee-to-go cups. The updated German version of GloboDiet provides about 3550 standard units, 100 photo series, about 50 different household measures, and 24 food shapes. Interviewers may enter individual recipes of the participants by specifying the description and quantity of each ingredient or using preset standard recipes and, if necessary, change single ingredients. The standard recipes have also been updated. New dishes, especially international dishes such as sushi, have been added, while other dishes which have lost popularity in Germany, such as those containing offal, were eliminated.

To assess the quality of the updated version including possible errors in its application and to build a basis for interpreting future study results, a validation study was conducted. The aim of the ErNst (‘Erfassung der Energie- und Nährstoffzufuhr’, engl. ‘assessment of energy- and nutrient intake’) study was to compare protein and potassium intake, estimated from GloboDiet food consumption data linked to the German Nutrient Database BLS (Bundeslebensmittelschlüssel), with nitrogen and potassium excretion in 24 h urine. Nitrogen and potassium are described as suitable validation factors [28]. Nitrogen excretion can be used to validate protein intake [28]. Potassium was used as it is considered a suitable biomarker for validation studies given its presence in a variety of foods, including vegetables and fruits [4]. The correlation between intake and excretion relies on the assumption that subjects are in a balance, meaning there is no accumulation or loss in their bodies [4,11].

Complementing the analyses above, sodium intake was compared with its urinary excretion as well as energy intake with total energy expenditure (TEE). Salt used during food preparation and added during mealtimes is difficult to quantify. A gap between estimated sodium intake and sodium excretion is expected [29,30,31]. To describe the extent of this deviation was a further aim of this study. Energy intake in comparison to energy expenditure is described as a surrogate measure when assessing the overall quality of dietary assessment methods [32]. Energy intake and expenditure underly daily fluctuations [33]. So, in this short-term analysis, it can only be used as a rough estimate of the agreement between intake and expenditure at the group level.

The hypothesis is that the updated German version of GloboDiet also provides valid estimates of nutrient intake. The different results are intended to provide a detailed insight into the quality of the updated German version of GloboDiet as it results from a short-term application.

## 2. Materials and Methods

### 2.1. Study Design

The ErNst study was conducted as a cross-sectional study at the Max Rubner Institut (MRI) in Karlsruhe, Germany, between October and December 2018 to validate the updated German version of GloboDiet. In order to obtain meaningful results, a necessary sample size of 50 men and 50 women was calculated. The test strength was calculated by the Leibnitz Institute for Social Science in Mannheim, Germany. A convenience sample was recruited by using an MRI-internal database, and announcements were made via the internet as well as local media. Only individuals were recruited whose nutrient or energy intake was not known to be affected by disease or medication. Details on the study design, as well as inclusion and exclusion criteria, can be found in Dötsch et al. [34]. Participants were informed about the overall study procedures and the purpose of the validation. A questionnaire was sent prior to the first visit to the study centre to the participants to gather general information about drug use and socio-economic information. On the first day at the study centre, participants handed in their questionnaires, and height and weight were measured to calculate body mass index (BMI, kg/m^2^). In addition, a bioelectrical impedance analysis (BIA) was performed to assess fat mass and fat-free mass. Participants were also fitted with an accelerometer to record their activity data and heart rate.

Urine collection and analysis of urine samples is described elsewhere [34]. On the second visit to the study centre, urine samples were handed over to the study team and the accelerometers were removed. The 24 h recall interviews to derive energy and nutrient intakes were conducted on the same day that the measurements with accelerometers and urine sampling took place.

### 2.2. Participants

**Figure 1:**
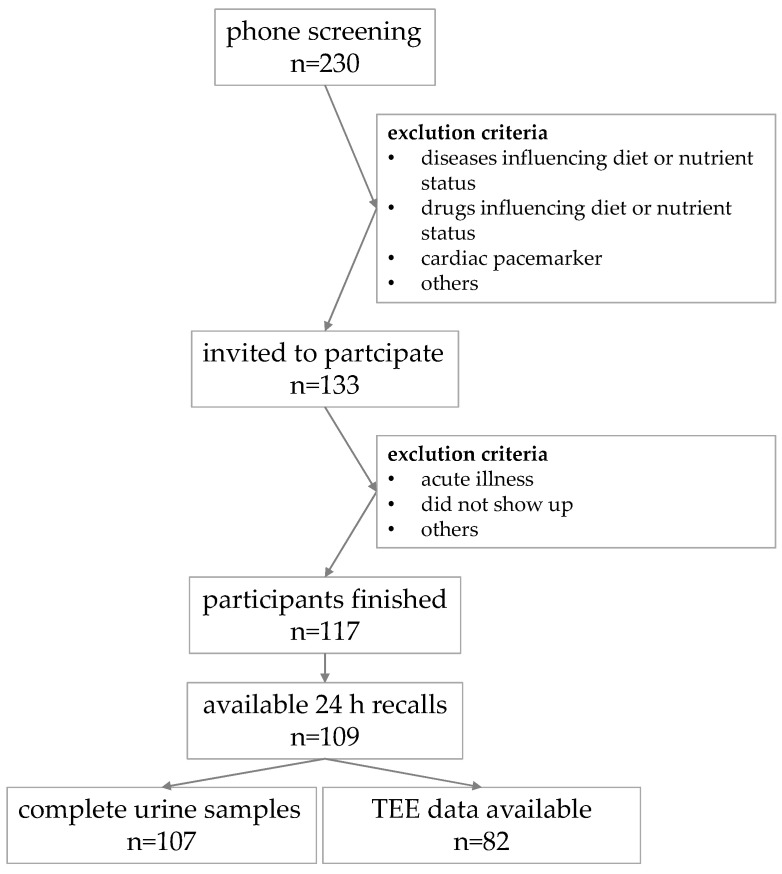
Flowchart describing the recruitment process and available data points of the ErNst sample modified by: [34].

The recruitment procedure as well as the exclusion criteria are presented in a flowchart in Figure 1. In total, the sample comprises available 24 h recall data from 109 participants (52 men, 57 women). Participants were equally distributed in the following age groups: 18–39, 40–59, and 60–79 years. For urine analysis, complete data from 107 participants (51 men, 56 women) were available. Urinary creatinine excretion was measured for a rough estimate of the completeness of 24 h urine samples, given the intra-individual variability in creatinine production and creatine or creatinine content in foods [28,35]. To test the completeness, the ratio between observed and estimated creatinine should be greater than 60% [36]:

Creatinine-ratio_women_ [%] = (100 × creatinine [mg/d])/(21 × weight [kg])

Creatinine-ratio_men_ [%] = (100 × creatinine [mg/d])/(24 × weight [kg]).

The mean creatinine quotient for men and women was 87% and 78%, respectively. Accordingly, all participants were included.

Physical activity was measured via accelerometers to calculate TEE. Measurements were lacking for 27 participants (9 men, 18 women), and 82 participants (43 men, 39 women) remained for comparison of energy intake and TEE.

### 2.3. Assessment of Food Consumption by 24 h Recalls (GloboDiet)

Owing to organisational constraints, 24 h recalls were either conducted on Mondays, Wednesdays, or Thursdays and thus covered food and beverage consumption on Sundays, Tuesdays, or Wednesdays, respectively. Participants were asked face-to-face by trained interviewers about the food and beverages as well as dietary supplements consumed the previous day. In the first step of the interview, general information such as participants’ sex and age were collected. To establish a so-called quick list, participants were asked chronologically using different food consumption occasions about all foods, beverages, and supplements consumed during the previous day. In the next step, single food items were described, quantified, and, if necessary, added. To assess the consumed amount of the food items, different quantification factors like density, edible parts, raw-to-cooked, and fat absorption factors are implemented in GloboDiet. In the last step, control questions and integrated quality checks were completed [14,23]. To calculate energy, protein, potassium, and sodium intake, the German Nutrient Database BLS version 3.02 was used [37]. For this step, single food items were manually linked on the basis of the assessed description to food items in the German Nutrient Database.

### 2.4. Nitrogen, Potassium, and Sodium Urine Excretion

In general, the proportion of nitrogen excreted in urine varies from 78% [38] and 80% [11,39,40] to 90% [41]. These differences are mainly explained by the influence of diet [41]. To estimate total nitrogen excretion in the ErNst study, the amount of nitrogen analysed in urine was divided by 0.78 and 0.90 as upper and/or lower limits, respectively. As protein consists of about 16% nitrogen [42], the lower and upper limits were multiplied by 6.25 to compare it with protein intake. By these means, upper and lower limits of excretion were calculated and subsequently compared to the calculated amounts from food consumption.

The proportion of potassium excreted in urine ranged from 77% [9,38] and 80% [40] to 90% [43]. The amount of potassium measured in the urine was therefore divided by 0.77 and 0.90 to calculate the upper and lower limits, respectively, and then to compare it with the calculated amounts from food consumption.

For sodium, the proportion from total food consumption excreted in the urine varies between 86% [11,44], 90 [29], and 95% [31]. Therefore, the amount of sodium excreted in urine was divided by 0.86 and 0.95 to calculate the upper and lower limits and subsequently compared to the amount from food consumption.

### 2.5. Energy Expenditure

The resting energy expenditure (REE) was calculated considering sex, age, fat-free mass, and fat mass [45]. The activity energy expenditure (AEE) was assessed using an accelerometer (ActiHeart from CamNtech) [46] for at least 24 h, at which acceleration data and heart rate were recorded. The dietary-induced thermogenesis (DIT) was set at 10% of the energy content of the foods consumed [47,48]. Total energy expenditure (TEE) was calculated with the following formula [45]:

TEE (kcal) = REE (kcal) + AEE (kcal) + DIT (10%).

In addition, physical activity level (PAL value) was calculated as the quotient of TEE and REE.

### 2.6. Statistical Analysis

Statistical analyses were performed using SAS (version 9.4) and R (version 4.2.3). Due to known differences in food consumption between men and women, e.g., shown in the German national nutrition survey 2 [15], all results were stratified by sex.

Neither food consumption nor nutrient intake variables were normally distributed. Measures of food consumption and nutrient intake are presented as arithmetic means, medians, standard errors, as well as 5th (P05) and 95th (P95) percentiles.

Different methods were applied to provide a comprehensive picture for the validation. Between-group differences were tested in SAS with Wilcoxon-signed rank tests and confidence intervals. Differences are considered to be significant at a level of *p* < 0.05 or if the confidence intervals do not overlap based on three decimal places. To describe the degree of agreement between intake and excretion/expenditure, the Spearman rank coefficient was calculated (also using SAS) [49].

The statistic program R version 4.3.1 was used with the package ‘blandr’ version 0.5.1 to present Bland–Altman plots [50]. Bland–Altman plots show the difference between pairs of measurements (here, between intake and excretion/expenditure) against the mean of both measurements. A Bland–Altman plot is therefore suitable for visualising measurement differences [51,52]. A horizontal centre line marks the mean of all individual differences between intake and excretion/expenditure. In ideal agreement, the mean difference between the pairs of measurements is zero. In addition, the two horizontal dotted lines named ‘limits of agreement’ mark the corresponding empirical standard deviation (mean ± 1.96 times) above and below the centre line. The in-between area covers 95% of the participants. The limits of agreement can be used to describe systematic or random errors, for example, a mean difference between two measurements that are consistently positive or negative. A clearly recognisable plot pattern could indicate an error [11,28,35]. Due to the small deviations of Bland–Altman plots with upper and lower calculated excretion limits, only plots for the upper limit from protein, potassium, and sodium excretion are presented.

## 3. Results

Table 1 shows the characteristics of the sample. The proportion of women and men, as well as the number of participants in each of the three age groups, was balanced. The sample had a high proportion of normal weight (60%), highly educated (76%), and non-smoking (91%) participants.

### 3.1. Protein and Potassium

Protein and potassium intake and excretion are shown in Table 2. Applying the lower urinary nitrogen excretion limit, no difference between GloboDiet-derived protein intake and urinary excretion was shown, neither for men nor women. If the upper urinary nitrogen excretion limit was assumed, intake and excretion differed significantly in men and women, with excretion exceeding intake.

For potassium, assuming the upper limit of excretion, a discrepancy between potassium intake and excretion was shown in men (excretion exceeding intake). In women, no difference between potassium intake and excretion was found.

Spearman rank correlation coefficients for protein were r = 0.62 (*p* < 0.001) for both men and women and for potassium they were r = 0.35 (*p* < 0.05) for men and r = 0.10 (n.s.) for women.

**Figure 2:**
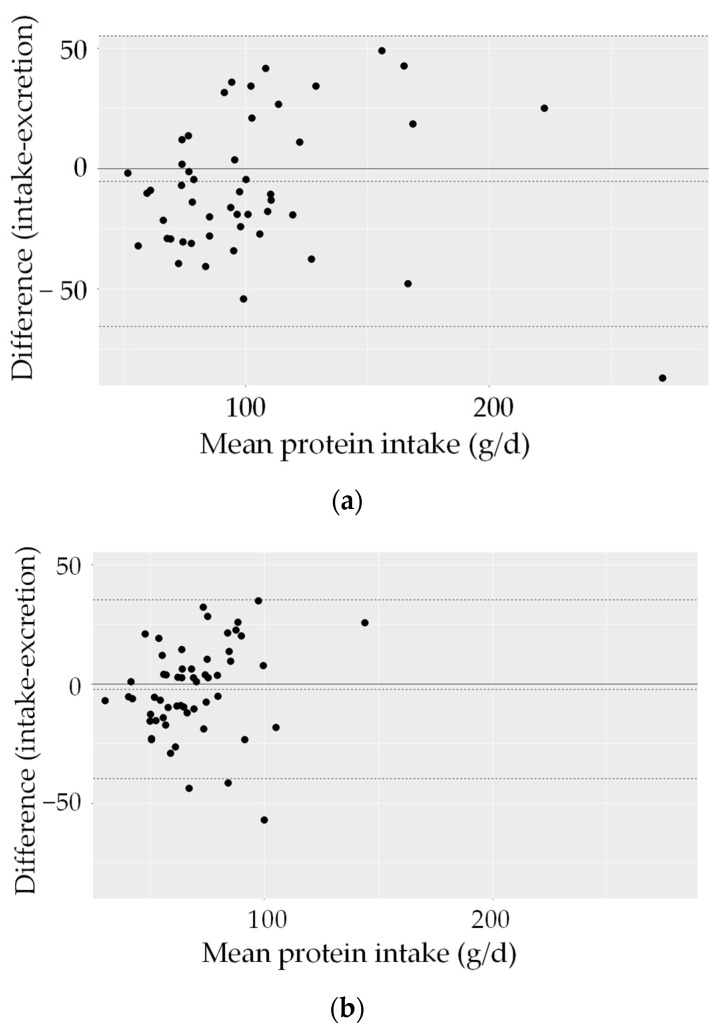

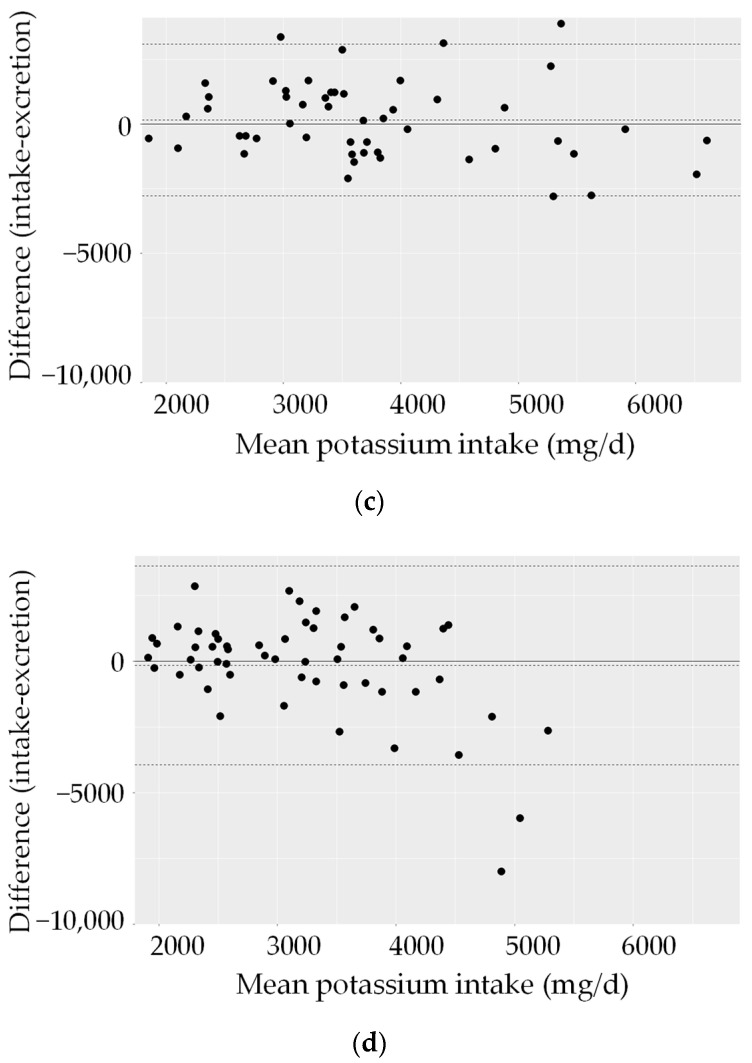
Individual differences between intake calculated from 24 h recalls and urine excretion plotted against the mean intake (Bland–Altman plot) (56 women, 51 men). Mean difference between intake and excretion (—); 2 SD limits of agreement (- - -). (**a**) Protein, men; (**b**) protein, women; (**c**) potassium, men; (**d**) potassium, women.

Bland–Altman plots for protein and potassium (Figure 2) show a good agreement between intake and excretion: The limits of agreement, in which 95% of the participants are included, indicate differences for protein between about ±50 g/day for men and ±40 g/day for women. For potassium, differences between approximately +3000/−2000 mg/d for men and approximately +1000/–5000 mg/d for women were found. The plots suggest a slight tendency for smaller differences in low intakes and larger differences in higher intakes.

### 3.2. Sodium

Irrespective of sex, sodium intake was lower than urinary sodium excretion (Table 3). This applies to the lower as well as to the upper limit. The Spearman correlation between sodium intake and excretion was r = 0.23 (n.s.) for men and r = 0.33 (*p* < 0.05) for women.

**Figure 3:**
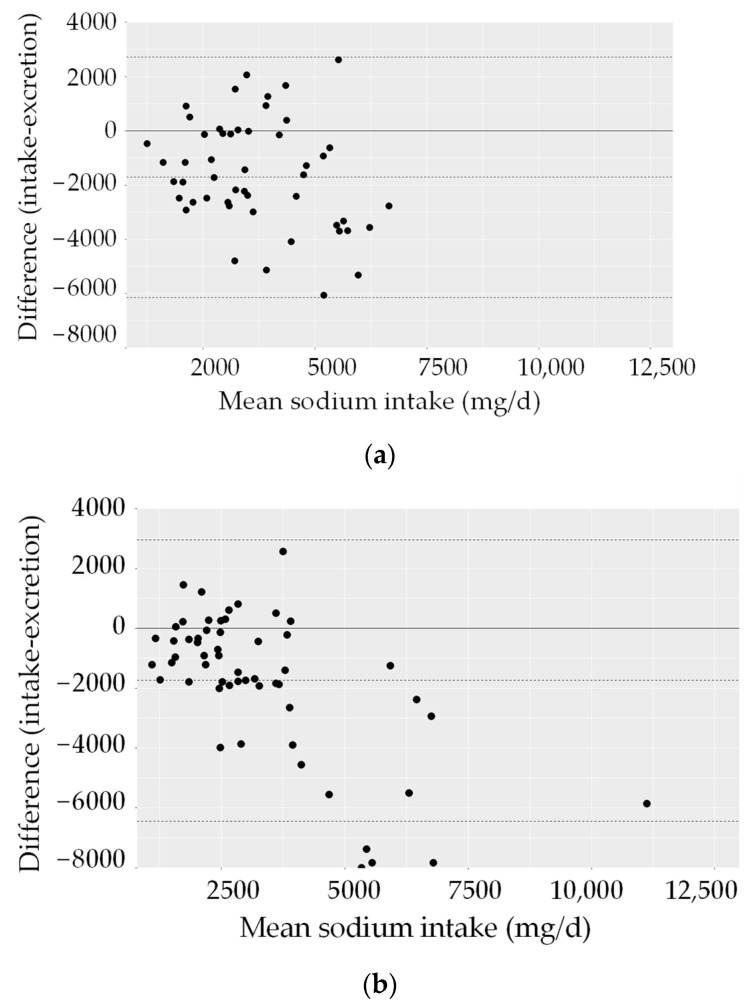
Individual differences between intake calculated from 24 h recalls and urine excretion plotted against the mean intake (Bland–Altman plots) (56 women, 51 men). Mean difference between intake and excretion (—); 2 SD limits of agreement (- - -). (**a**) Sodium, men; (**b**) sodium, women.

The low intake compared to excretion is also seen in the negative values of the corresponding Bland–Altman plots (Figure 3). The limits of agreement show differences for men of approximately +2000/−6000 mg/d and differences for women of approximately +3000/−8000 mg/d.

### 3.3. Energy

Energy intake and energy expenditure are presented in Table 4. There was no difference between reported energy intake and TEE for both men and women. The correlation coefficient between energy intake and TEE was r = 0.35 (*p* = n.s.) for men and r = 0.06 (*p* < 0.05) for women.

**Figure 4:**
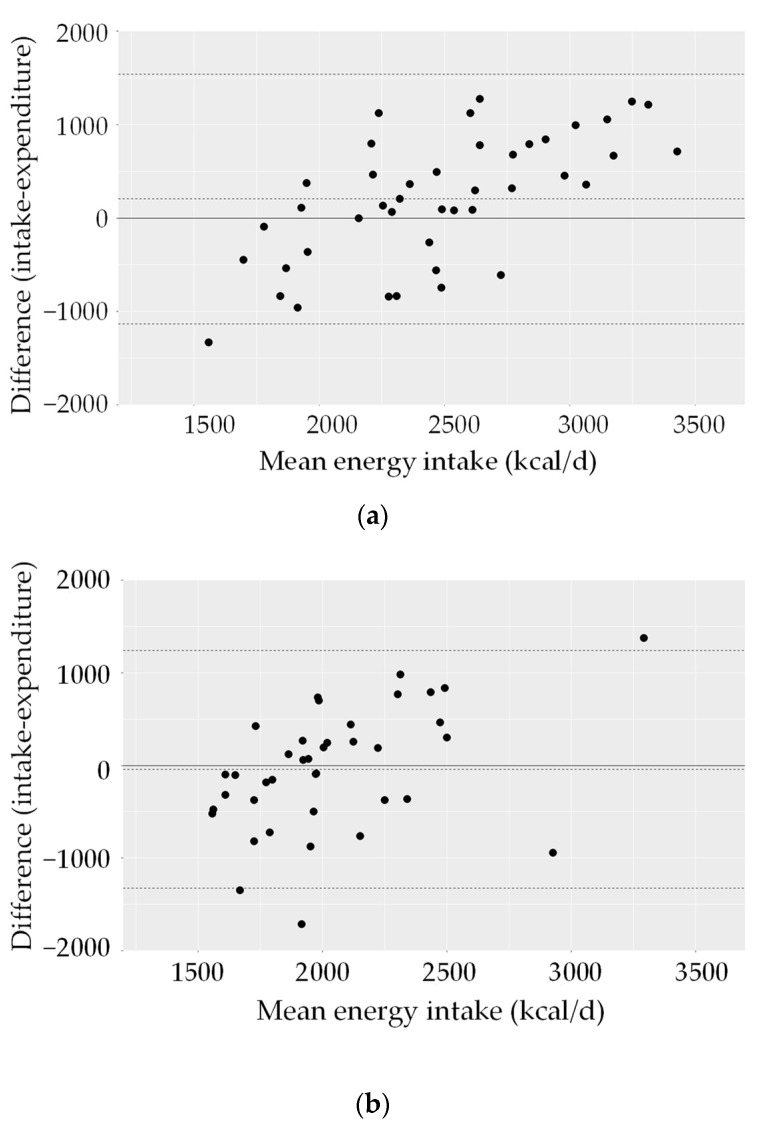
Individual differences between intake calculated from 24 h recalls and TEE plotted against the mean intake (Bland–Altman plot), among adults (56 women, 51 men). Mean difference between intake and expenditure (—); 2 SD limits of agreement (- - -). (**a**) Energy, men; (**b**) energy, women.

Figure 4 shows the corresponding Bland–Altman plots for energy intake and expenditure. The limits of agreement were approximately +1500/−1000 kcal/d in men and approximately ±1200 kcal/d in women. Regardless of sex, participants with lower energy intake reported lower energy intake compared to TEE and participants with higher energy intake reported higher intake compared to TEE.

## 4. Discussion

The aim of the present study was to validate an extensively updated German version of GloboDiet within the framework of the ErNst study. The objective of a validation study of a dietary assessment instrument is to understand the errors when interpreting the results [2,3]. For this purpose, in the presented study, nutrient intake was compared with the corresponding measured physiological parameters as well as energy intake with calculated total energy expenditure.

Protein and potassium are well-known biomarkers for validating dietary assessment instruments [38]. However, in validation studies, it is presupposed that participants have no accumulation or loss of nutrients [4,11]. Due to the exclusion criteria applied during recruitment, a protein balance can be assumed for most participants. Nevertheless, variations such as diet, influencing excretion, cannot be completely ruled out in this study. To consider different diet-dependent excretion rates of the nutrients [41], the comparison between intake and excretion included estimated values for a lower and an upper limit. For the presented results, it was not known if the upper or the lower limit was more accurate for the specific study sample. Therefore, it can only be seen as an approximation.

If the lower limit of excretion was assumed, no difference between intake and excretion was seen. If the upper limit was assumed, differences between protein intake and excretion suggesting an underestimation of protein intake could be found. However, the Bland–Altman plots show a good agreement and the Spearman rank correlation coefficients were higher than 0.6. Kuhnle [11] described that if using a single-day sample only, generally a correlation coefficient between protein intake and excretion of approximately 0.5 can be assumed. It can be concluded that the updated German version of GloboDiet provides valid estimates of protein intake, but an underestimation at the group level might be possible. The results of the correlation coefficients are in line with those of others. Crispim et al. [21], who validated EPIC-Soft in five European study centres, found correlations for protein intake and excretion between 0.42 and 0.65. Slimani et al. [24] compared results of 24 h recalls with EPIC-Soft from 12 study centres in six European countries and derived ratios of reported to excreted nitrogen between 0.54 and 0.99. But, they also reported an underestimation of nitrogen intake. An underestimation was also reported in the US Observing Protein and Energy Nutrition (OPEN) study. They assessed the structure of dietary measurement error in 24 h recalls and showed an underestimation of protein intake of 11–15% [53]. Again, similar to the present results, Koch et al. reported an underestimation of protein intake of 10% and a correlation of 0.66 using 24 h online recalls [54]. So, presented results of protein intake and excretion are in line with those of other studies.

Similarly to protein, if the lower limit of excretion was assumed for potassium, no difference between intake and excretion was seen. If the upper limit was assumed, an underestimation of potassium intake was seen for men but not for women. The Bland–Altman plots indicate a good agreement, but the correlation coefficients between potassium intake and excretion were weak in men (0.35) and in women (0.16). Altogether, differences between intake and excretion were more pronounced for potassium compared to protein. An underestimation of potassium intake using 24 h recalls has been previously reported [2,21,29]. Crispim et al. [21] conducted a validation of EPIC-Soft versions in five European countries. They found correlation coefficients ranging from 0.31 to 0.69, with an underestimation of potassium intake (except for results from Czech Republic suggesting an overestimation [21]). In the representative US survey, the National Health and Nutrition Examination Survey (NHANES) which utilized one or two 24 h recalls, an underestimation of potassium intake was observed, too [29]. In a review where five large validation studies were pooled, correlations of potassium intake and excretion of 0.43 (men) and 0.39 (women) were reported [2]. However, in another validation study drawing on online 24 h recall data, a correlation coefficient of 0.46 and no significant difference between intake and excretion were found [54]. Even though the results described in the literature so far are inconsistent, there seems to be a tendency towards underestimation of intake, as described in the present study. The present results of potassium intake and excretion are in the range of those of the EPIC-Cohort, where different country-specific EPIC-Soft versions and country-specific food composition databases were used [21]. One possible reason for the weak potassium correlation in the present study may be the single-day data collection because high correlation coefficients are to be expected for multiple excretion measurements [40]. Yet, it remains unclear why the correlation between potassium intake and excretion is rather weak despite similar potassium intake and excretion values compared to other studies.

The present study further aims to describe the magnitude of the gap between sodium intake and excretion. Considerably lower sodium intake compared with sodium excretion was found in men (37–43%) and women (42–47%). In addition, correlation coefficients were weak in men (0.23) and women (0.34). In a review, five large validation studies were pooled. They also derived weak correlations of sodium intake and excretion, 0.39 for men and 0.24 for women [2]. In a review by McLean [55], including 20 studies using 24 h recalls, correlations ranged from 0.16 to 0.72. In all studies also, an underestimation of sodium intake was reported. In contrast, Freedman et al. reported an underestimation of only approximately 30% of sodium intake compared to excretion.

As described by Campino et al., sodium intake originates from different sources [56]. It is well known that a substantial amount of sodium intake comes from salt in food preparation [30,31,57]. There are different possible reasons for the underestimation of sodium intake in this study. Reasons can be an underestimation of the salt content in cooked foods, salt added at the table, as well as different salt amounts in composite foods like bread, dishes like pizza, or foods from the out of home sector. The used Nutrient Database also possibly contributes to the underestimation. The used Nutrient Database (BLS version 3.02) does not include all those possible amounts of salt added during meal preparation (informal information, MRI, 2023), a general issue when using food composition databases [29]. In addition, in Nutrient Databases, values for nutrients are calculated as average values, that do not necessarily correspond to the individual consumed food. Considering these issues, dietary assessment instruments are assumed to underestimate sodium intake. This has also been reported by others [29,30,31]. Estimating sodium intake via 24 h urine samples is considered as a gold standard for estimating a population’s sodium intake [11,30]. As described by Kuhnle [11], a single sample of urinary sodium is strongly associated with intake, but an average urinary output based on several samples would give better results, because the sodium excretion follows a long-term cycle.

Energy intake is an important factor in interpreting the results of dietary assessment instruments, to the effect that the misreporting of food consumption results in energy misreporting and, therefore, in the misreporting of accompanying nutrients. Energy intake can be seen as a surrogate measure of total food consumption [12,32]. Therefore, the difference in energy intake and TEE was analysed at a group level. At the group level, there was no difference between reported energy intake and TEE for men and women. But, there was a weak correlation between energy intake and TEE in men (0.35) and even more in women (0.06). Contrarily, the Bland–Altman plots show a good agreement. For the estimation of energy intake, TEE was assessed on the same day as the 24 h recalls, but only for one day. The presented ratios of energy intake to TEE should be interpreted with caution, as energy intake may be balanced over more than one day [1,12,13]. However, at the group level, the present results seem to fit quite well. Also, Freedman et al., who pooled five large validation studies, concluded that when using only one 24 h recall, the correlation between energy intake and TEE was weak and rose when three more recalls were conducted [40].

## 5. Strengths and Limitations

A strength was that, similar to the study population to be studied within the upcoming survey of the German National Nutrition Monitoring, the ErNst-study includes a mixed-sex adult sample including different age groups. Due to organisational limitations, the convenience sample was mainly recruited from an own-participant database. The sample was usually known for conscientious study participation. This was repeatedly seen in earlier studies biased towards a healthier dietary and physical activity behaviour compared to the general population. As a consequence, it should be noted that the sample was not aimed to be representative of the German population and the present results cannot be generalized.

A further limitation was that only one 24 h recall per person and one 24 h urine sample for the same day were covered. Day-to-day variations are unaccounted for. The correlation between intake and excretion tends to be higher when using repetitive 24 h recalls [2].

Given the conscientious sample, creatinine concentration was used as a rough estimate for the completeness of urine samples. Para-amino benzoic acid (PABA) as a possible method to check the completeness of 24 h urine samples was not used in this study. When returning urine samples, participants were interviewed about problems in the urine sampling procedure and excluded in case of any problems.

A strength of this validation study is the calculation of TEE on the basis of acceleration measurements. However, doubly labelled water, the gold standard when assessing human energy requirements [32,58], was not applied due to cost limitations.

Because of different excretion factors, described in the literature, an upper and a lower limit for the excretion of protein, potassium, and sodium were calculated. The real excretion factor for the study sample was unknown.

## 6. Conclusions

In the present study, biochemical biomarkers as well as different statistical analyses were applied to provide a comprehensive view about the validation of the updated German version of GloboDiet.

Taking all results together, protein and potassium intakes are validly assessed by the updated German version of GloboDiet. Sodium intake was underestimated by approximately more than a third, which should be considered when interpreting results of salt intake. To deal with this, different options are possible. In addition to the 24 h recalls, a (food frequency) questionnaire can be applied to assess further and detailed information about salt use. Also, more differentiated information about salt used in food preparation could be added to the used Nutrient Database. The results underline the need to determine sodium excretion in urine in addition to the dietary assessment, preferably over several days. Although only one day was assessed, the mean energy intake showed no difference against the total energy expenditure and thus seemed to fit quite well at a group level. Despite the partly ambiguous results, the updated German version of GloboDiet if linked to the German Nutrient Database provides valid estimates of nutrient intake.

## Figures and Tables

**Table 1:** Characteristics of the ErNst sample.

	All	Men	Women
Participants (n (%))	109 (100)	52 (47.7)	57 (52.3)
Age (years) (mean)	49.6	50.3	49.0
Descriptive percentages	% ^1^	% ^1^	% ^1^
Age (years)			
<40	33	33	33
40–59	32	33	32
≥60	35	35	35
Body mass index (kg/m^2^)			
<25	60	50	68
25, 0–29, 9	33	44	23
≥30, 0	7	6	9
School education			
Low (<10 years)	8	6	8
Intermediate (10 years)	17	13	19
High (>10 years)	76	81	72
Smoking status			
Non-smokers	91	92	89
Occasional smokers	6	4	7
Smokers	4	4	4

^1^ Differences of 100% are due to rounding.

**Table 2:** Protein and potassium intake and excretion (mg/d), stratified by sex.

Protein Intake and Excretion	Mean ^1^	CI (Mean) ^2^	Std Error	P05	P50	P95	Diff
**Men (n = 51)**							
Protein intake	101	88/114	6	53	91	223	
Protein excretion, lower limit	106 _n.s._	94/119 _n.s._	6	65	99	190	−5
Protein excretion, upper limit	123 *	109/137 _n.s._	7	75	114	220	−22
**Women (n = 56)**							
Protein intake	68	61/74	3	39	64	103	
Protein excretion, lower limit	70 _n.s._	64/75 _n.s._	3	41	68	114	−2
Protein excretion, upper limit	80 *	74/87 *	3	47	78	132	−12
Potassium intake and excretion							
**Men (n = 51)**							
Potassium intake	3848	3502/4194	172	2085	3754	6282	
Potassium excretion, lower limit	3692 _n.s._	3276/4109 _n.s._	207	1843	3417	6927	156
Potassium excretion, upper limit	4316 *	3829/4802 _n.s._	242	2154	3993	8096	−468
**Women (n = 56)**							
Potassium intake	3145	2893/3397	126	1836	2999	4687	
Potassium excretion, lower limit	3295 _n.s._	2871/3719 _n.s._	211	1503	2817	6599	−150
Potassium excretion, upper limit	3851 _n.s._	3356/4346 _n.s._	247	1757	3293	7713	−706

n.s. = not significant; * = significant differences; Diff = difference between intake and excretion. ^1^ Significant difference between intake and excretion (as indicated by Wilcoxon rank test *p* values < 0.05). ^2^ Significant difference between intake and excretion (non-overlapping confidence intervals).

**Table 3:** Sodium intake and excretion (mg/d), stratified by sex.

Men (n = 51)	Mean ^1^	CI (Mean) ^2^	Std Error	P05	P50	P95	Diff
Sodium intake	2889	2481/3297	203	812	2714	5185	
Sodium excretion lower limit	4598 *	4018/5178 *	289	1948	4170	8224	−1709
Sodium excretion upper limit	5079 *	4438/5720 *	319	2152	4606	9085	−2190
Women (n = 56)							
Sodium intake	2406	2032/2780	190	922	2118	5290	
Sodium excretion lower limit	4151 *	3416/4886 *	367	1493	3436	9470	−1745
Sodium excretion upper limit	4585 *	3773/5397 *	405	1649	3796	10,461	−2179

Diff = Difference between intake and excretion. * = significant differences; ^1^ Significant difference between intake and excretion (as indicated by Wilcoxon rank test *p* values < 0.05). ^2^ Significant difference between intake and excretion (non-overlapping confidence intervals).

**Table 4:** Energy intake and energy expenditure (kcal/d), stratified by sex.

Men (n = 43)	Mean ^1^	CI (Mean) ^2^	Std Error	P05	P50	P95	Diff
Energy intake	2580	2354/2806	112	1434	2542	3786	
Resting metabolic rate	1755	1715/1794	20	1518	1788	1919	
Activity energy expenditure	396	339/453	28	164	352	731	
Total energy expenditure	2388 _n.s._	2298/2479 _n.s._	45	1948	2373	2850	−192
Women (n = 39)							
Energy intake	2019	1827/2211	95	1060	1953	2910	
Resting metabolic rate	1347	1293/1401	27	1150	1316	1550	
Activity energy expenditure	494	406/581	43	210	411	1017	
Total energy expenditure	2047 _n.s._	1926/2167 _n.s._	60	1634	1934	2753	28

n.s. = not significant; Diff. = difference between intake and TEE. ^1^ Significant between energy intake and TEE (as indicated by Wilcoxon rank test *p* values < 0.05). ^2^ Significant between energy intake and TEE (non-overlapping confidence intervals).

## Data Availability

In accordance with the informed consent of the ErNst (Erfassung der Energie- und Nährstoffzufuhr) participants, pseudonymised study data could be provided to external researchers upon request for cooperation or data-use purposes. In this case, a cooperation or data-use agreement must be concluded in advance. The use of ErNst study data is therefore possible with a detailed prior justification of its scientific purpose and after agreement with the MRI. However, informed consent would, in general, allow the publication of a publicly available data set of anonymised study data, which is also planned in the future [34].
